# S06 Engaging peers, parents and pupils to increase physical activity among adolescents

**DOI:** 10.1093/eurpub/ckac093.027

**Published:** 2022-08-29

**Authors:** Maria O'Kane, Marie H Murphy, Angela Carlin, Alison Gallagher

**Affiliations:** 1 Centre for Exercise Medicine, Physical Activity and Health, Sports and Exercise Sciences Research Institute, Ulster University, Derry, United Kingdom; 2 Nutrition Innovation Centre for Food and Health (NICHE), Ulster University, Coleraine, United Kingdom

**Keywords:** Adolescents, peers, parents, school-based interventions

## Abstract

**Background:**

Regular physical activity is associated with physiological and mental health benefits for adolescents including improved fitness and cardiometabolic health, increased muscle and bone strength and reduced risk of obesity. Despite this, globally, many adolescents (81%) fail to meet physical activity guidelines. Physical activity levels decline as children move into adolescence and through to adulthood and may affect the likelihood of developing chronic health conditions. It is recognised that parental support and friendship networks play an important role in attenuating declines in physical activity during adolescence and it is vital that we develop effective interventions to help adolescents stay active.

**Aim:**

This symposium will engage policymakers, professionals, scientists and stakeholders to discuss research projects on Engaging peers, parents and pupils to increase physical activity among adolescents. The goals of this symposium are to highlight the challenges and ongoing work to address sub-optimal levels of physical activity in this population and disseminate the results of novel physical activity interventions to develop knowledge and understanding. This symposium will share experiences, learning and best practice in Patient and Public Involvement (PPI), transitioning from formative research to feasibility testing and upscaling interventions and it will also allow for debate and to identify gaps and priority areas for physical activity among adolescents.

**Symposia presentations:**

1. Putting young people at the heart of physical activity research design: The Walking In ScHools (WISH) Study (Professor Marie Murphy, Ulster University)

This presentation will outline the importance of youth PPI and demonstrate how PPI can be embedded within research design using the WISH Study Youth Advisory Group as a case study.

2. The Girls Active Project (GAP): Co-design and feasibility of an after school-based physical activity intervention (Sara McQuinn, Dublin City University)

This presentation will discuss the factors influencing physical activity behaviour in adolescents, outline how using public and patient involvement (PPI) and the behaviour change wheel provided a framework for developing the Girls Active Project, and the results from our feasiblity trial.

3. Supporting our lifelong engagement: mothers and teens exercising (SOLE MATES); from formative research to feasibility testing (Dr Elaine Murtagh, University of Limerick).

Dr Murtagh will discuss the value of formative research in developing interventions and present findings from a novel mother-daughter multi-component physical activity programme.

4. Co-production of the Move Well, Feel Good movement behaviours intervention (Stuart Fairclougt, Edge Hill University)

This presentation will describe phase 1 of the Move Well, Feel Good study, which aimed to co-produce and evaluate the feasibility of a primary school physical activity intervention to improve children’s motor competence and mental health.

5. The Walking In ScHools (WISH) Study: Development and evaluation of a peer-led school-based walking intervention in adolescent girls from pilot to fully-powered trial (Professor Marie Murphy, Ulster University)

Professor Murphy will describe how the WISH pilot study guided the development of a full trial to evaluate the effects of a peer-led walking intervention at increasing physical activity in adolescent girls.

